# Serum neurofilament indicates that DBS surgery can cause neuronal damage whereas stimulation itself does not

**DOI:** 10.1038/s41598-022-05117-x

**Published:** 2022-01-27

**Authors:** Anika Frank, Jonas Bendig, Iñaki Schniewind, Witold H. Polanski, Stephan B. Sobottka, Heinz Reichmann, Katja Akgün, Tjalf Ziemssen, Lisa Klingelhoefer, Björn H. Falkenburger

**Affiliations:** 1 Department of Neurology, University Hospital Carl Gustav Carus, Dresden, Germany; 2 German Center for Neurodegenerative Diseases (DZNE), Dresden, Germany; 3 Department of Neurosurgery, University Hospital Carl Gustav Carus, Dresden, Germany; 4 Department of Neurology, Center of Clinical Neuroscience, Technische Universität Dresden, Dresden, Germany; 5 Department of Neurology, Technische Universität Dresden, University Hospital Carl Gustav Carus, Dresden, Germany

**Keywords:** Parkinson's disease, Biomarkers, Basal ganglia

## Abstract

Deep brain stimulation (DBS) is a potent symptomatic therapy for Parkinson’s disease, but it is debated whether it causes or prevents neurodegeneration. We used serum neurofilament light chain (NFL) as a reporter for neuronal damage and found no difference between 92 patients with chronic STN-DBS and 57 patients on best medical treatment. Serum NFL transiently increased after DBS surgery whereas the initiation of STN stimulation did not affect NFL levels, suggesting that DBS surgery can be associated with neuronal damage whereas stimulation itself is not.

## Introduction

Deep brain stimulation of the subthalamic nucleus (STN-DBS) is an effective symptomatic treatment for Parkinson's disease (PD). In addition, neuroprotective effects of chronic DBS have been suggested by preclinical animal studies^1^. Potential mechanisms include reduced glutamatergic excitotoxicity^2^, enhanced synaptic remodeling and increased release of neurotrophic factors promoting survival of dopaminergic neurons^3–5^.

Clinical studies, however, could not confirm a disease-modifying effect. Although STN-DBS-treated patients show a sustained symptomatic benefit and a prolonged survival^6^, PET and postmortem analyses revealed the same continuous decline in dopaminergic neurons as in patients without DBS^7,8^. Patients with STN-DBS can develop long-term adverse events such as cognitive^9^ and psychiatric deterioration^10^. Consequently, advanced age, cognitive deficits, and active psychiatric disorders constitute contraindications for DBS surgery^11^. It has remained unclear, however, whether unfavorable outcomes of STN-DBS result primarily from advanced disease, from the surgical procedure or from STN stimulation itself.

For a broader understanding of these effects, blood biomarkers that report neurodegeneration can provide valuable insight. Neurofilament light chain (NFL), a structural protein expressed exclusively in neurons, has emerged as such a biomarker. With the convenient possibility to detect neuronal damage in blood samples, NFL has been used in a variety of neurological conditions to assess disease activity and evaluate treatment efficacy^12^. In PD, higher levels of NFL correlate with disease severity^13^ while still being significantly lower compared to atypical parkinsonian syndromes, consistent with the more rapid disease progression in these conditions^13–15^.

The objective of this study was to determine whether and to what extent DBS influences serum NFL in patients with PD. We therefore measured serum NFL longitudinally in patients submitted to DBS (a) before and after DBS surgery and (b) before and after acute STN stimulation. In addition, we collected samples from patients on chronic STN stimulation and compared them with patients on long-term best medical treatment.

## Results and discussion

Demographic and clinical data are summarized in Table 1. DBS-treated patients showed significantly higher disease duration than patients on best medical treatment (p < 0.0001, Mann–Whitney-U) and more severe signs of disease (Hoehn & Yahr, p = 0.0011; MOCA scores, p = 0.0033, Mann–Whitney-U)—as expected from patients with an advanced therapy like DBS. There was no significant difference in age, levodopa equivalent daily dose (LEDD), UPDRS III scores and AIMS between patients on best medical treatment and chronic STN-DBS.

**Table 1:** Demographic and clinical data.

	Best medical treatment (n = 57)	Chronic STN-DBS (n = 92)	Patients submitted to STN-DBS: preoperative baseline (n = 18)
Age, years	66 (58–70)	67 (60.8–71.2)	63 (56–69)
Sex, male/female, n (male, %)	34/23 (59.7)	67/25 (72.8)	12/6 (71.4)
Disease duration, years	6 (4–9)	15 (12–20)	9 (6–10)
Motor subtype tremor/akinetic-rigid/equivalent, n	13/28/16	18/30/43	5/6/7
Hoehn & Yahr stage	2 (2–3)	3 (2–3)	2 (2–2)
LEDD, mg	725 (515–1150)	676.5 (478–962.9)	1115 (795–1408)
UPDRS III score (on medication and on stimulation where applicable)	22 (16–28)	21 (15–31.5)	22 (18–30)
AIMS score	0 (0–1.5)	0 (0–5)	0 (0–10)
MOCA score	27 (26–29)	24 (22–27)	28 (27–29)
DBS duration, years		5.5 (3.2–8.4)	
Serum NFL, pg/ml	15.9 (11.7–21.1)	20.7 (13.1–30.2)	14.8 (10.3–18.1)

Data are median (Q1: lower quartile—Q3: upper quartile) except for sex and motor subtype (n).

*LEDD* levodopa equivalent daily dose, *UPDRS III* Unified Parkinson's Disease Rating Scale Part III, *AIMS* Abnormal Involuntary Movement Scale, *MOCA* education-adjusted Montreal Cognitive Assessment, *DBS* Deep Brain Stimulation, *NFL* Neurofilament Light Chain.

### NFL in patients with chronic DBS and patients with best medical treatment

**Figure 1:**
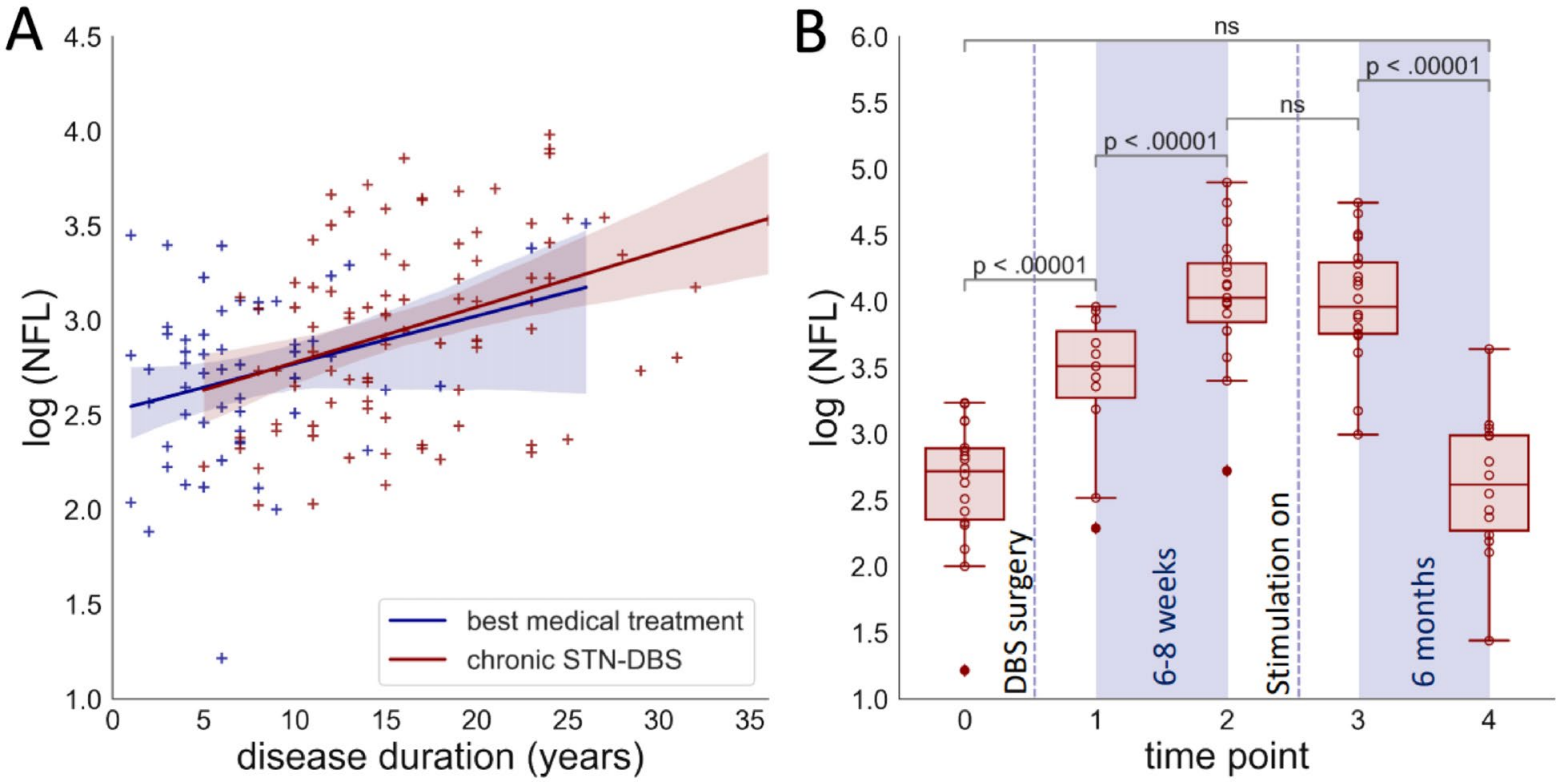
(**A**) Serum NFL increases with disease duration, but not through STN-DBS. NFL values were not normally distributed and therefore plotted as logNFL vs. disease duration. Markers represent PD patients with chronic STN-DBS (red) and on best medical treatment only (blue). Lines represent linear regressions with slopes of 0.029 and 0.025, intercepts of 2.49 and 2.52. (**B**) Longitudinal NFL measurements. Serum NFL was determined in individual patients at 5 time points before and after DBS surgery: 0: baseline/preoperative, 1: 3–5 days postoperative (stim off), 2: 6–8 weeks postoperative (stim off), 3: 3–5 days after testing and activating the stimulation (stim on), 4: 8 months after DBS implantation (stim on). Values for individual patients and values for complete datasets only are depicted in Supplemental Figs. S1 and S2. Longitudinal clinical data are shown in supplemental Table S1.

To determine the effect of STN-DBS on neurodegeneration in PD patients, we compared serum NFL in 149 patients recruited in our movement disorders clinic. 92 patients were on chronic STN-DBS (Fig. 1A, red markers) and 57 received best medical treatment only (Fig. 1A, blue markers). In both groups, NFL increased with age (Spearman ρ = 0.5, p < 0.0001), Hoehn & Yahr stage (ρ = 0.44, p < 0.0001), lower MOCA scores (ρ = − 0.43, p < 0.0001), disease duration (ρ = 0.41, p < 0.0001), and UPDRS part III scores (ρ = 0.35, p < 0.0001), which is consistent with previous findings by others^13,16^. We did not observe a systematic difference between male and female patients in our cohort. After correcting for disease duration and age, we found no difference in serum NFL between the two groups (p = 0.36, ANCOVA).

To find the chief determinants of serum NFL levels in our sample, we fitted linear models to the factors with the highest correlation coefficients. The best linear model included age, MOCA score and disease duration and explained 46% of logNFL variance (Multiple R^2^ = 0.46).

### Longitudinal measurements of NFL and the effect of stimulation

Serum NFL varied widely between individuals (Fig. 1A). In order to determine the effect of DBS more precisely, we measured serum NFL longitudinally in 21 individual patients at five time points before and after surgery (Fig. 1B). In our center, there is a 6–8-week delay between the implantation of DBS electrodes and the initiation of STN stimulation. In routine care, this delay allows for neurological testing without the microlesion effect. In the context of this study, it enabled the discrimination between effects of the DBS surgical procedure and effects of STN stimulation.

Indeed, serum NFL increased significantly between the baseline before surgery (median = 14.8 pg/ml (10.3 – 18.1 pg/ml)) and the first time point after surgery (3–5 days postoperatively median = 33.5 pg/ml (26.5—43.8 pg/ml)). This increase most likely results from the surgical procedure. However, due to the small sample size, we could not identify any predictor for a particularly steep postoperative increase in NFL. Specifically, there was no significant influence of the operation mode (awake vs. general anesthesia, p = 0.24, Mann–Whitney-U), the number of inserted microelectrodes (Spearman ρ = 0.44, p = 0.078) or baseline NFL (median split, Mann–Whitney-U, p = 0.82). Although neuronal damage has not been formally demonstrated during DBS surgeries, the concept is commonly accepted—as evidenced by the “microlesion effect”, which describes the immediate reduction in PD symptoms after surgery even without electrical stimulation^17^. Interestingly, serum NFL continued to increase between the early postoperative time point and the 6–8 week time point (median = 56.1 pg/ml (46.8–72.9 pg/ml)). A similar increase in extent and time course was observed with comparable interventions like intraventricular catheter implantation representing serum NFL dynamics after a defined surgical brain trauma^18^.

Six to eight weeks after surgery, DBS was tested extensively and stimulation was established. There was no significant effect of DBS testing and initiation of stimulation on serum NFL (median NFL at time point 3 = 52.55 pg/ml (42.8–73.2 pg/ml)). Prolonged STN stimulation did not alter NFL levels and NFL values even decreased after the initiation of stimulation and returned to baseline levels 8 months after surgery (median NFL at time point 4 = 13.8 pg/ml (9.7–19.9 pg/ml)). The missing increase of serum NFL after electric stimulation of the STN is consistent with the lacking difference between patients with chronic STN-DBS and patients on best medical treatment indicating that electric stimulation does not cause neuronal damage (Fig. 1A).

## Conclusion

We observed a transient increase in serum NFL after implantation of DBS electrodes, which peaked 6–8 weeks after surgery and declined to baseline values 8 months after surgery. This increase likely reflects the known microlesion effect and NFL dynamics. The observed transient neuronal damage confirms current clinical practice to avoid DBS surgery in vulnerable patients, in particular elderly or demented patients. In contrast, STN stimulation did not elicit an increase in serum NFL—neither the acute initiation of stimulation nor chronic stimulation. This finding suggests that STN stimulation does not lead to neuronal damage and confirms current clinical practice not to turn off DBS in patients with advanced PD, i.e. in stages where surgery for DBS is no longer recommended. Limitations of our study include the reliance on a single center and a single marker for neurodegeneration. The relatively small sample for the perioperative group did not allow us to reliably determine predictors of perioperative NFL increase. Such an analysis could be helpful to better select patients for DBS surgery and should be addressed in a larger population of patients recruited at more than one center.

## Methods

### Study population and design

Patients were recruited at the University Hospital Dresden between December 2018 and April 2021. The study was approved by the institutional review board of the Technische Universität Dresden (EK533122019, EK 487122016). Written informed consent was obtained from all participants before inclusion in the study. All experiments were performed in accordance with relevant guidelines and regulations.

The study includes three different cohorts of PD patients: (A) 57 patients on best medical treatment, (B) 92 patients on chronic DBS treatment, and (C) longitudinal measurements in 21 patients undergoing DBS surgery. Serum NFL for groups A and B was measured once while NFL for group C was measured on five consecutive time points. For the longitudinal measurements, the following numbers of blood samples were available: (i) within 30 days before surgery (n = 18), (ii) 3–5 days postoperatively (n = 11), (iii) 6–8 weeks postoperatively, before activating STN stimulation (n = 19), (iv) 3–5 days after activating STN stimulation (n = 20) and (v) 8 months after DBS implantation (n = 14). The differing numbers result from organizational issues such as treatment in another facility before or after surgery. A “spaghetti plot” showing the NFL time course in individual patients is included as Supplemental Fig. S1.

Clinical and demographic data were collected prospectively. This included information about motor and cognitive status (Hoehn & Yahr stage, UPDRS part III, Montreal cognitive assessment (MOCA)) and the presence of disease-related complications. For group A the same data was obtained, but transferred from patients’ records. Full datasets were available, except for MOCA in group A (n = 34). Patients received implants of the following manufacturers: 51 Medtronic, 19 St. Jude / Abbott, 40 Boston Scientific.

### Serum NFL measurements

Serum samples were stored at − 20 °C after preparation. NFL measurement was performed as described previously^19,20^, using the Advantage NF-Light Singleplex-Kit on a Simoa HD-1 instrument (Quanterix). Calibrators and diluted serum samples were measured in duplicates. The lower threshold of quantification was 0.775 pg/ml. Both the mean intraassay coefficient of variation of duplicates and the mean interassay coefficient of variation were < 10%.

### Statistical analyses

To normalize the right-skewed distribution of NFL natural log-transformation (logNFL) was used, as described by others^16^. Comparisons between groups were carried out using the Mann–Whitney-U-Test adjusted with Bonferroni correction. An ANCOVA with logNFL as dependent variable was used to compare treatment groups while adjusting for age and disease duration. The results were comparable when adjusting for disease duration only. ANCOVA assumptions were assessed by inspection of linear regressions, ANOVA for variable interaction, Shapiro–Wilk-test and Levene’s test on residuals. Correlations between logNFL and clinical scores were assessed with Spearman's rank test. To assess the unique contributions of parameters to logNFL, a linear model was used.

To compare longitudinal NFL measurements, we fitted a linear mixed model to predict logNFL with time points and subjects. Time points were compared by ANOVA and post-hoc testing was performed by paired t-tests of estimated marginal means with Bonferroni correction.

Statistical analyses were performed with R-Studio or Python. Sample size was based on the size of our patient cohort and not determined by a sample size estimate.

## Supplementary Information

Supplementary Information.
