# Supplementary material for: Serum neurofilament indicates that DBS surgery can cause neuronal damage whereas stimulation itself does not
Source: Sci Rep. 2022 Jan 27;12:1446. doi: 10.1038/s41598-022-05117-x (PMC8795190; doi:10.1038/s41598-022-05117-x)
Supplement: Supplementary file 1 — Supplementary Information. [file 41598_2022_5117_MOESM1_ESM.pdf]

Supplemental Material for

Serum Neurofilament Indicates That DBS Surgery Can Cause Neuronal Damage Whereas Stimulation Itself Does Not

Anika Frank MD[1,2], Jonas Bendig MD[1], Iñaki Schniewind[1], Witold H. Polanski MD[3], Stephan B. Sobottka MD[3], Heinz Reichmann MD[1], Katja Akgün MD[4], Tjalf Ziemssen MD[4], Lisa Klingelhoefer MD*[1], Björn H. Falkenburger MD*[1,2]

* These authors contributed equally.

1) Department of Neurology, University Hospital Carl Gustav Carus, Dresden, Germany

2) German Center for Neurodegenerative Diseases (DZNE), Dresden, Germany

3) Department of Neurosurgery, University Hospital Carl Gustav Carus, Dresden, Germany

4) Center of Clinical Neuroscience, Department of Neurology, Technische Universität Dresden, Germany

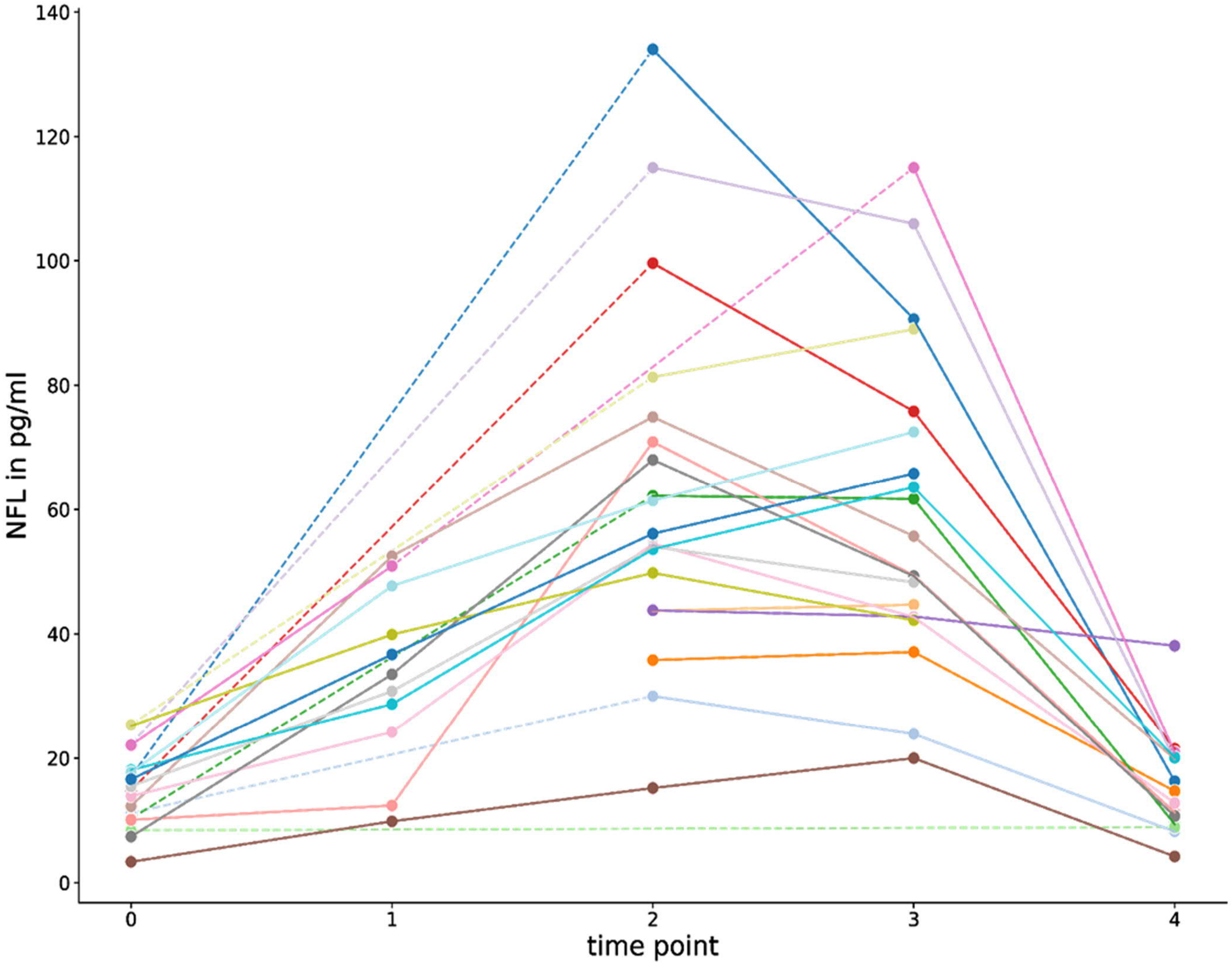

Supplementary Figure S1: Serum NFL was determined in individual patients at 5 time points before and after DBS surgery: 0: baseline/preoperative, 1: 3-5 days postoperative (stim off), 2: 6-8 weeks postoperative (stim off), 3: 3-5 days after testing and activating the stimulation (stim on), 4: 8 months after DBS implantation (stim on). Lines represent the trajectories for individual patients. Solid lines represent intervals with complete data, while dashed lines connect intervals with missing data points.

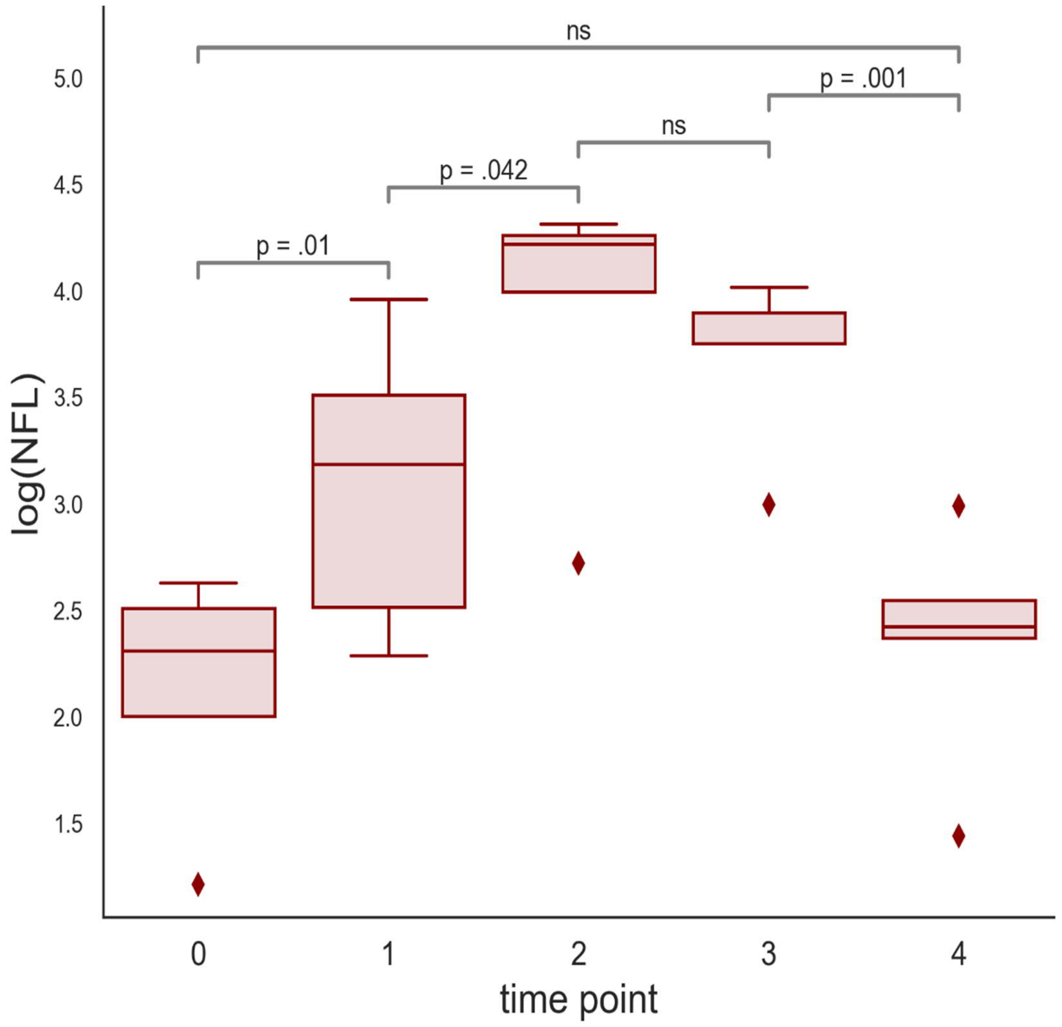

Supplementary Figure S2: Log (NFL) for patients at the five time points before and after DBS surgery only displaying patients with complete datasets (n=6). Repeated measures ANOVA with posthoc paired t-test and correction for multiple testing (false discovery rate, Benjamini-Hochberg), revealed significant differences between time point 0 vs. 1, 1 vs. 2 and 3 vs. 4. There was no significant change in serum NFL after initiation of electric stimulation (time point 2 vs. 3) and NFL levels returned to baseline level 8 months after DBS implantation (0 vs. 4).

Supplementary Table 1 Clinical data for longitudinal measurements for patients undergoing DBS surgery at time points before surgery, 2 months after surgery and 8 months after surgery.

	Time point 0 (preoperative baseline)	Time point 3 (2 months postoperative)	Time point 4 (8 months postoperative)
LEDD, mg	1115 (795-1408)	404 (210-607)	502 (300-867)
UPDRS III score (on medication and on stimulation where applicable)	22 (18-30)	18 (13-20)	13.5 (10.75-18.25)
AIMS score	0 (0-10)	0 (0-0)	0 (0-5)
MOCA score	28 (27-29)	27 (23-28)	28 (26-29)
Serum NFL, pg/ml	14.8 (10.3-18.1)	55.7 (42.7-89.0)	14.55 (10.08-19.83)

Clinical data and serum NFL for patients undergoing DBS surgery at three consecutive time points. The table only contains data from the 18 patients that have a complete dataset at baseline examination (as in Figure S2). Age: 63 (56-69) years, sex: 12 male / 6 female, disease duration 9 (6-10) years, motor subtype: 5 / 6 / 7 patients with tremor-dominant / akinetic-rigid / equivalent type, devices: 4 / 14 St. Jude/Abbott / Boston Scientific. Data are median (Q1: lower quartile - Q3: upper quartile). Abbreviations: LEDD, levodopa equivalent daily dose; UPDRS III, Unified Parkinson's Disease Rating Scale Part III; AIMS, Abnormal Involuntary Movement Scale; MOCA, education-adjusted Montreal Cognitive Assessment; DBS, Deep Brain Stimulation; NFL, Neurofilament Light Chain.
